# “It's because they care”: understanding pathways to classroom concentration problems among HIV-affected children and youth in Western Kenya

**DOI:** 10.1080/09540121.2016.1159651

**Published:** 2016-07-08

**Authors:** Morten Skovdal

**Affiliations:** ^a^ Department of Public Health, University of Copenhagen, Copenhagen, Denmark

**Keywords:** HIV, concentration, education, HIV-affected children, ethics of care, Kenya

## Abstract

Children and young people living in households affected by HIV are experiencing poorer educational outcomes compared to their peers. This article explores how different forms of marginalisation interface and manifest themselves in classroom concentration problems, undermining their education. This mixed qualitative methods study was conducted with teachers and pupils from three primary and three secondary schools in the Siaya County of Western Kenya. Specifically, it involved 18 teachers through individual interviews and 51 HIV-affected children and youth through individual interviews (*n* = 47) and Photovoice (*n* = 51). Verbatim transcripts were imported into NVivo10 for thematic indexing and analysis. The analysis revealed three core pathways to classroom concentration problems amongst HIV-affected pupils. One, a general ‘lack of care’ and neglect in the context of household poverty and illness, meant that many of the participating pupils went to school hungry, unable to follow classes. Others were teased by peers for looking visibly poor, and felt anxious when in school. Two, some HIV-affected pupils play a key role in keeping their household afloat, generating food and income as well as providing practical support. ‘Caregiving’ pupils often reported coming to school exhausted, with limited physical and mental energy left for learning. Three, many participating pupils had their minds at home (‘caring about’). They were concerned about sick or frail household members, thinking about their next meal and care needs. Although the pupils demonstrated an admirable attentiveness to the needs of others, this came at a heavy price, namely their ability to concentrate in class. The paper argues that care ethics, household poverty and familial HIV are central to understanding the classroom concentration problems of HIV-affected pupils. To ensure school-going children and youth affected by HIV have the same opportunities as their peers, education initiatives must simultaneously alleviate both household poverty and other challenges pertaining to familial HIV.

## Introduction

Education provides marginalised children and youth of the HIV epidemic with a window of hope and opportunity for a future free from poverty, disease and disparity (Meinert, 2009). However, children and youth who face unfair and avoidable deprivation of basic care and resources, often as a result of a combination of household poverty and familial HIV, face significant disparities when it comes to their schooling (Orkin, Boyes, Cluver, & Zhang, 2014). The impact of AIDS on children's education was most profound at the beginning of the millennium. Countries like Swaziland and the Central African Republic experienced a 20–36% decline in school enrolment due to AIDS and orphanhood, with girls most affected (UNAIDS, 2002). A decade on, and despite the growing availability of antiretroviral therapy, children of the HIV epidemic continue to experience poor educational outcomes. A literature review of 23 studies exploring children's educational outcomes found overwhelming evidence that HIV-affected children are less likely to be enrolled in school, or less likely to attend school regularly if enrolled (Guo, Li, & Sherr, 2012). Studies have also identified children affected by HIV to have worse school performance relative to their peers (Skovdal, Webale, Mwasiaji, & Tomkins, 2013; Tu et al., 2009).

These education disparities, combined with disenfranchisement pertaining to health, psychological well-being and social status (Sherr et al., 2014), lock many children and youth affected by HIV into paths that prevent them from yielding many of the long-term benefits of education, such as employment, a healthier start to life for their own children, voice and civic engagement, and health-enabling behaviours, including defence against HIV (UNICEF, 2009). To ensure children and youth affected by HIV have an equitable chance in life to pursue a future of their desire, we must continue to unpack, understand and address the underlying causes and consequences of their marginalisation, including education inequities.

Much research into education inequities focus on school enrolment, attendance, correct-grade-for-age and performance, and reveal a complex web of interrelated factors and variables that are associated with poor educational outcomes for HIV-affected children and youth. For instance, at the level of the family, children who have lost their mother are less likely to attend school, or attend regularly if enrolled, be in the correct-grade-for-age, or perform well compared to other orphaned or non-orphaned children (Birdthistle et al., 2009; Evans & Miguel, 2007; Nyamukapa & Gregson, 2005). A recent cohort study from Zimbabwe examined the impact of HIV on the education of different groups of HIV-affected children (Pufall et al., 2014). The study found that a child's HIV status has no significant bearing on his or her education. Being an orphan on the other hand, whether maternal, paternal or double, increased the odds of not being in the correct-grade-for-age, or simply not completing primary school. The study also found that being a young carer was associated with poor school attendance (Pufall et al., 2014).

A much less researched educational outcome pertains to classroom concentration problems. This is despite the fact that a South African survey has found 42% of adolescents living in AIDS-affected households to report that concentration at school was difficult, attributing this to their concern and worry about sick relatives (Cluver, Operario, Lane, & Kganakga, 2012). A recent study by the same team found no direct association between familial HIV and school enrolment, attendance, correct-grade-for-age and concentration problems. Instead, they found poverty and internalising problems to indirectly catalyse the negative impact of orphanhood and caregiver sickness on adolescents’ educational outcomes, including their ability to concentrate in class (Orkin et al., 2014). Also Tu et al. (2009) have found a link between poor mental health and classroom concentration problems amongst HIV-affected children and youth. Although a few qualitative studies have in passing reported on classroom concentration problems among HIV-affected children and youth with caregiving roles (Cluver et al., 2012; Evans, 2012; Skovdal & Ogutu, 2009; Watkins, Sello, Cluver, Kaplan, & Boyes, 2014), no previous study has set out to explicitly explore the phenomenon of classroom concentration problems as experienced by HIV-affected children and youth. Building on previous work, this paper sets out to unpack and understand how familial HIV, poverty, internalising problems and care ethics interface and manifest themselves in classroom concentration problems, undermining the education of children and youth.

## Methods

This paper draws on qualitative data from a phenomenological study that aimed to explore the school experiences of children and youth affected by HIV. The study was conducted in 2012, funded by the Norwegian Research Council. Ethics approval was granted by the Norwegian Social Science Data Services (27655/AH/RF) and The National Council for Science and Technology in Kenya (NCST/RCD/12A/012/043B). Informed and written consent was obtained from all participants. To respect confidentiality assurances, pseudonyms have been used throughout.

### Study location and participants

The study took place in the Siaya County of Nyanza Province. The county has a population of approximately 880,000 people, of which nearly half live below the poverty line (GoK, 2013). Most people rely on small-scale subsistence farming, growing maize, kale and tomatoes, as well as breeding livestock and poultry. The county has experienced some of the highest HIV prevalence rates in Kenya and currently has a prevalence rate at 17.8%, three times the national average of 5.6% (NACC, 2014). The study was conducted in collaboration with a local educational NGO, which helped identify three rural, low-resource and high-HIV-prevalence communities, each serviced with both a primary and secondary school. At each community, contact was made with the schools, the local administration (chief, assistant chief and elders) and a local women's group to inform them of the study and to ask for their endorsement and support. The stakeholders were welcoming and two members from each woman's group volunteered to serve as community guides.

A total of 26 primary school pupils (aged 10–17, mean age of 13.5 years), 25 secondary school pupils (aged 15–20, mean age of 16.5) and 18 teachers participated in the study (see Table 1). Through snowball and maximum variation sampling, the community guides identified and invited 60 orphaned and vulnerable pupils to participate in the study. The criteria used for their selection included attending the local primary or secondary school, and being either orphaned, presumably by AIDS, or living with a sick or elderly relative. Three pupils declined to participate due to disinterest, and a further six dropped out after having completed an assessment questionnaire, citing time constraints as the reason. Recruitment of teachers was done by the research team and based on their interest and availability to participate.

**Table 1.** Research methods used with the sample of participants.

Participants and data collection methods	Primary school (age 10–17)		Secondary school (age 15–20)		Total
	Male	Female	Male	Female	
Orphaned and vulnerable pupils (*n* = 51)					
Photovoice	12	14	12	13	51
In-depth interviews	10	10	14	13	47
Teachers (*n* = 18)					
In-depth interviews	6	3	3	6	18

### Data generation and analysis

Data for this study were generated through two qualitative methods: photovoice and semi-structured individual interviews (see Table 1). Due to language limitations of the Caucasian lead investigator (M.S.), data were generated with the support of three local, and Dholuo-speaking, researchers. The local researchers were carefully selected and trained by M.S. to conduct research with children.

Photovoice was chosen as a method to provide the pupils with a fun and participatory platform for them to identify, represent and enhance their perspectives of their lifeworlds (Skovdal & Cornish, 2015; Wang & Burris, 1997). In an initial workshop, study objectives were discussed and pupils were shown how to use a disposable camera and potential ethical challenges of taking pictures in the communities were also discussed. One key challenge identified by the participants related to the ethics of taking pictures of their bed-ridden relatives. It was agreed that participants finding it problematic to take a picture of a scenario would instead make a drawing. The participants had two weeks to capture through pictures *what life is like for school-going orphaned and vulnerable children* as well as *what supports them through their education.* At a second workshop, the participants received two copies of all the pictures: one copy for them to keep and a copy for them to work with in the project. At the workshop, they were invited to select six pictures they were happy to share with the research team, showing: (i) something or someone in school who makes them happy or sad; (ii) something or someone who helps them attend school; (iii) something they lack, need or wish they had more of to improve their education or (iv) the importance of education. Participants were then invited to write a short story to each of the pictures, responding to three questions: Why do you want to share this picture? What is the story that this picture tells? How does this story relate to your life and/or the lives of other children in your school? A total of 224 photos and 119 drawings, each with a written reflection, were generated from this exercise.

Interviews with the pupils included themes such as their care and living arrangements, experiences of going to school as well as their perspectives on the support potential of teachers and schools. Teachers were asked about the challenges facing orphaned and vulnerable children, including the implications of these challenges to their educational outcomes, as well as their responses to these challenges. All interviews were digitally recorded, translated and transcribed. Stories written in Dholuo were also translated. Transcripts and pictures were imported into NVivo 10 for thematic network analysis (Attride-Stirling, 2001). This paper reports on eight emerging basic themes that speak to three pathways (organising themes) to classroom concentration problems (global theme). Table 2 outlines the thematic network and structure guiding the results section.

**Table 2.** Thematic network analysis: from basic themes to global theme.

Basic theme	Issues discussed in codes	Organising themes	Global themes
Neglect and lack of basic necessities	Impoverished home environments Lack of adult care	‘Lack of care’ leaves children and youth lethargic and anxious	Pathways to poor school concentration
Go to school with empty stomachs	Food is a prerequisite for learning Lack of food at home Cannot concentrate because of empty stomachs		
Poor social relations	Children are teased by peers Family in-fights and disagreements		
Pre-occupied keeping the family afloat by offering practical support	Busy providing care for sick or frail household members Work to generate food and income Life is stressful and hectic	‘Caregiving’ leaves children and youth exhausted	
Physically and mentally exhausted from caregiving	Feeling tired Lack of energy		
Concerned about the needs of members in their household	Worry about a sick or frail household member Wonder how they can best help the household member Fear the household member needs help	‘Caring about’ household members preoccupies their mind	
Mind focused on preparing the next meal	Households depend on their economic contribution Stress about the next meal for their household		
Fear the death of a loved one	Worry they will return home to find their loved one deceased Have images of loved ones suffering and dying		

## Results

### ‘Lack of care’ leaves children and youth lethargic and anxious

Accounts from both teachers and pupils suggest that many of the most vulnerable HIV-affected children and youth do not receive adequate care from their guardians, leaving them anxious about when their next meal is, or how they are going to pay for their school fees. As the account by 15-year-old Millicent suggests, such worries, not uncommonly, divert attention away from what is going on in the classroom, and may lead to school drop-outs.

There are times where a teacher may think that you are concentrating, yet your mind is not there. You worry that may soon to be sent home for fees, yet there is no money. You may also want to do your homework in the evening, yet there is no paraffin. There is also no food. This may trouble you so much. All this put together can enable one to lose hope and drop from school or go to school but without peace of mind. (Millicent, age 15, secondary school)

Lack of food was not only a worry experienced by many of the pupils. It was also reported to cause a physiological reaction of tiredness and lethargy in class. Pupils spoke about going to school on “empty stomachs” and how this affected their ability to concentrate. Teachers reported often observing the most vulnerable pupils half asleep, ascribing this to either exhaustion (from caregiving, as discussed later) or hunger. Samuel, an 18-year-old secondary school pupil, describes the link between having an empty stomach and falling asleep.

I may come to school, but while in class, when we are given something to do, I just feel very weak. This is because I may go to school without having eaten anything and feel hungry. So instead of reading, I sleep in class. (Samuel, age 18, secondary school)

Pupils living in households affected by HIV and poverty were said to look visibly poor, leaving them vulnerable to stigma and bullying from their peers. This, and the inability of some HIV-affected pupils to build supportive social relationships, was also reported to affect their concentration in class.

Maybe the uniform the child is wearing is torn and she feels embarrassed. This too can affect her studies as she may decide not to go to school because of her torn dress. She may be teased because of their parent's illness. This can prevent children affected by HIV from concentrating well. (Catherine, age 41, primary school teacher)

These references highlight that household poverty and familial HIV diminish the ability of some adults to care for their children. This in turn leaves some pupils anxious and lethargic, preventing them from concentrating in class. The references also suggest that these challenges – in some instances – may lead to school drop-outs.

### ‘Caregiving’ leaves children and youth exhausted

The dire home situation of many of the participating pupils, coupled with the limited ability of their sick or elderly guardian to cater for their needs, meant that many of the pupils took on significant caregiving roles and responsibilities. Several teachers, including Susan, claimed that young caregiving affected their ability to concentrate in class.

It is the responsibility of the child to bring food on the table, find medicines for her mother. If the mother is bedridden, the child may need to clean the mother. This child will not concentrate in class since she does a lot of work within a short period of time. (Susan, age 33, primary school teacher)

The pupils gave a little more detail on how caregiving can affect classroom concentration. Lucy, for example, spoke about how excessive caregiving leaves her mentally exhausted.

**Figure 1.**
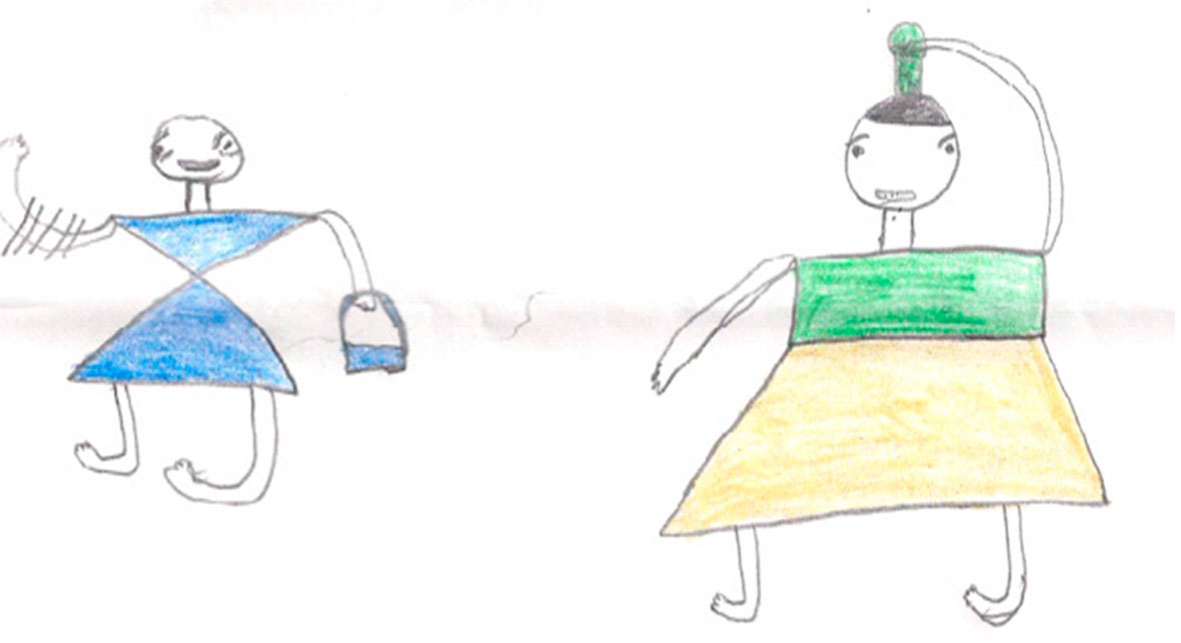
Drawing by Lucy.

The drawing (see Figure 1) shows me from school, I fetch firewood on my way back home and I carry them together with my bag. After my arrival back home, I run to get water; from there I struggle to find vegetables for supper. This makes me weak and have unstable mind. I'm thinking a lot. (Lucy, age 15, primary school pupil)

Lillian, speaking about one of her friends, exemplifies the physical exhaustion that some pupils with excessive home duties experience.

A friend of mine has to weed the garden in the morning before going to school. This makes him come to school late and very tired. Such children will sleep in class. (Lillian, age 15, primary school student)

### ‘Caring about’ household members preoccupies their mind

A third and prominent pathway to classroom concentration problems pertains to the pupils “caring about” relatives and significant household members. Many references were made to pupils from households affected by poverty and HIV as “stressed” or “unsettled” because of their attentiveness to the needs of others. Jane notes how she spends a lot of time in class thinking and worrying about her sick mother, whilst Beatrice is concerned about what her siblings will eat for their next meal.

I am providing care for my mother; I know very well that she is sick. When I am in class I am just thinking about her. I will be thinking about how she is coping without me around. Children who do not care will not worry and is just comfortable in class. (Jane, age 17, secondary school pupil)

I sometimes lack concentration in class when I think about what my siblings will eat. This mostly happens when we completely have nothing and can end up sleeping hungry not knowing what tomorrow holds for us. During such times I get disturbed a lot. (Beatrice, age 15, primary school pupil)

Void of daily contact and updates on the well-being of struggling relatives left boarding school pupils particularly concerned – a concern that for some like Judith translated into guilt for not being at home to help out:

Sometimes when I am seated in class I think about home and even find myself falling sick. Whenever my sister and brother visit me at school they tell me what is happening at home. They may tell me how they have not had supper or breakfast. I sometimes ask myself: Why do I have to eat here in school and get satisfied while they are starving? I can decide not to go and get food from the school kitchen. I don't deserve. (Judith, age 16, secondary school pupil)

Fear of losing a loved one was also reported to undermine the ability of some pupils to concentrate in class. For some pupils, it was the aging of their grandmother, whilst for others it was the deterioration of their AIDS-sick parents, as explained by Christine, a primary school teacher.

Children who are affected by this disease [HIV], may be born bright, but all of a sudden they start dropping in class. This is because of the many thoughts that they have in their heads. They think that their parent might die and …  …  … he now becomes less steady in class. This is really affecting them. (Christine, age 28, primary school teacher)

The circumstances surrounding this fear differed significantly between pupils. For Macrine, whose mother is HIV positive, it was the threat of parental suicide that occupied her mind in class.

Sometimes when my mum tells me that she will poison herself it becomes hard to concentrate in class because I think of home and my mom a lot. (Macrine, age 14, primary school pupil)

The link between ‘caregiving’, ‘caring about’ and classroom concentration problems appears to be gendered. As reflected above, most references on this topic were made by female pupils and teachers, highlighting the gendered nature of care work in many parts of rural sub-Saharan Africa (Evans, 2014; Evans & Thomas, 2009)

## Discussion

This paper set out to unpack and understand how familial HIV, poverty, internalising problems and caregiving interface and manifest themselves in classroom concentration problems. Echoing observations in South Africa (Cluver et al., 2012), the results of the analysis reveal that a general ‘lack of care’ and neglect in the context of household poverty and illness meant that many of the participating pupils went to school hungry, unable to follow classes. Others were teased by peers for looking visibly poor or for being associated with HIV, and felt anxious when in school. Household poverty and familial HIV also meant that many of the participating pupils played a key role in keeping their household afloat, generating food and income as well as providing practical and nursing support. Such ‘caregiving’ was often reported to leave the pupils exhausted, with limited physical and mental energy left for learning. Although being in school did provide HIV-affected pupils with a much needed break from caregiving, their minds were often reported to still be at home, “caring about” the next meal and care needs of sick or frail household members. In other words, the pupils' attentiveness to the needs of others (‘caring about’), and sense of responsibility (‘taking care of’) and ability (‘caregiving’) to care indicates an “ethics of care” (cf. Tronto, 1993 , pp. 131–136), which, in the context of household poverty and familial HIV (‘lack of care’), contributes to classroom concentration problems via internalising problems (worry, anxiety, concern) and physiological reactions (tiredness, exhaustion, lethargy).

This study has shown that the internalising problems or physiological reactions that contributed to classroom concentration problems for the HIV-affected pupils participating in this study are intrinsically linked to household poverty and familial HIV – highlighting their disfranchisement and inequitable disposition compared to their non-affected peers. Both the ‘lack of care’ from significant adults and the “ethics of care” exhibited by the pupils in this study were a direct result of living in a household affected by poverty and HIV. Efforts to reduce the educational disparities facing children from poor-resource and high-HIV-prevalence communities must not consider children's education in isolation from their home and care arrangements. The results of this study point to the need for *combination social protection* programmes that consider children and youth's livelihoods needs, parenting support needs as well as their educational needs, also referred to as “cash, care and classroom” by Cluver et al. (2015). A community-based capital cash transfer programme in Western Kenya, which provides community groups with funds to set up social enterprises, has demonstrated its potential to both strengthen livelihoods, further children's education, as well as strengthen orphan care and support structures at household and community levels (Skovdal, Mwasiaji, Webale, & Tomkins, 2011; Skovdal et al., 2013).
